# A new perspective on PTSD symptoms after traumatic vs stressful life events and the role of gender

**DOI:** 10.1080/20008198.2017.1380470

**Published:** 2017-11-13

**Authors:** Lisa J. M. van den Berg, Marieke S. Tollenaar, Philip Spinhoven, Brenda W. J. H. Penninx, Bernet M. Elzinga

**Affiliations:** ^a^ Institute of Psychology, Clinical Psychology Unit, Leiden University, Leiden, The Netherlands; ^b^ Department of Psychiatry/EMGO Institute for Health and Care Research, VU University Medical Center, Amsterdam, The Netherlands

**Keywords:** PTSD, aetiology, gender, traumatic events, life events, película de trauma, trastorno por estrés postraumático, TEPT, reconsolidación, intrusiones, memoria intrusiva, memoria involuntaria, imágenes mentales, memoria de trabajo, PTSD, 生态, 性别, 创伤事件, 生活事件

## Abstract

**Background**: There is an ongoing debate about the validity of the A1 criterion of PTSD. Whereas the DSM-5 has opted for a more stringent A1 criterion, the ICD-11 will leave it out as a key criterion.

**Objective**: Here we investigated whether formal DSM-IV-TR traumatic (A1) and stressful (non-A1) events differ with regard to PTSD symptom profiles, and whether there is a gender difference in this respect.

**Method**: This was examined in a large, mostly clinical sample from the Netherlands Study of Depression and Anxiety (*n *= 1433). Participants described their most bothersome (index) event and were assigned to either an A1 or non-A1 event group according to this index event.

**Results**: Remarkably, in men PTSD symptoms were even more severe after non-A1 than A1 events, whereas in women symptoms were equally severe after non-A1 and A1 events. Moreover, while women showed significantly higher PTSD symptoms after A1 events than men (29.9 versus 15.4% met PTSD criteria), there was no gender difference after non-A1 events (women: 28.2%; men: 31.3%). Furthermore, anxiety and perceived impact were higher in women than men, which was associated with PTSD symptom severity.

**Conclusion**: In sum, while women showed similar levels of PTSD symptoms after both event types, men reported even higher levels of PTSD symptoms after non-A1 than A1 events. These findings shed a new light on the role of gender in PTSD symptomatology and the clinical usefulness of the A1 criterion.

## Abstract (es)

**Planteamiento**: Los recuerdos intrusivos de trauma son un síntoma clave del trastorno por estrés postraumático (TEPT), por lo que interrumpir su recurrencia es muy importante. El desarrollo de las intrusiones fue obstaculizado por intervenciones visuoespaciales administradas hasta 24 horas después del trauma analógico. Se desconoce si las intervenciones pueden ser aplicadas posteriormente, y si la modalidad o la carga de la memoria de trabajo son factores cruciales.

**Objetivos**: Este estudio intenta probar: 1) si una tarea visuoespacial daría lugar a menos intrusiones en comparación con un grupo solamente de reactivación cuando se aplicara después de la reactivación del recuerdo 4 días después de la exposición al trauma analógico (replicación extendida); 2) si ambas tareas)es decir, una dirigida a ser visuoespacial, una más verbal) darían lugar a menos intrusiones que el grupo solamente de reactivación (efecto de la intervención), y 3) si la supuesta modalidad de la tarea (visuoespacial o verbal) es un componente crítico (efecto de la modalidad).

**Método**: Cincuenta y cuatro participantes fueron asignados aleatoriamente a reactivación + Tetris (visuospatial), reactivación + juegos de palabras (verbales), o sólo reactivación (sin tarea). Vieron una película aversiva (día 0) y registraron recuerdos intrusivos de la película en el diario A. El día 4, se reactivó el recuerdo, después de lo cual los participantes jugaron al Tetris, a juegos de palabras, o no hicieron ninguna tarea durante 10 minutos. Luego escribieron en un segundo diario (B). Las hipótesis informativas fueron evaluadas utilizando los factores de Bayes.

**Resultados**: Reactivación + Tetris y reactivación + juegos de palabras resultaron en relativamente menos intrusiones desde el último día del diario A hasta el primer día del diario B que solo reactivación (objetivo 1 y 2). Por tanto, ambas tareas fueron eficaces incluso cuando se aplicaron días después del trauma analógico. La reactivación solo no fue efectiva. Reactivación+juegos de palabras parecen resultar en menos intrusiones que Reactivación+Tetris (objetivo 3, efecto de la modalidad), pero esta evidencia fue débil. Los análisis exploratorios mostraron que los juegos de palabras eran más difíciles que el Tetris.

**Conclusiones**: La aplicación de una tarea 4 días después de la película del trauma (durante la reconsolidación de la memoria) fue eficaz. La modalidad versus el problema de la carga de la memoria de trabajo no es concluyente.

## Abstract (zh)

**背景**：对PTSD诊断A1标准的讨论一直持续进行，DSM-5使用了更严格的A1标准，ICD-11则将其从关键诊断标准中删除。

**目标**：我们在此考察了过去的DSM-IV-TR创伤性（A1）和压力性（非A1）事件在PTSD症状剖面（symptom profiles）上的不同，以及是否存在性别差异。

**方法**：在一个大型的主要是临床样本的关于抑郁和焦虑的荷兰研究中（n=1433），被试描述了他们最受困扰的（指标）事件，并据此划分出A1或者非A1事件组。

**结果**：显著的差别是，男性在非A1事件后的PTSD症状比A1事件后更严重，而女性在非A1事件和A1事件后的症状程度相等。并且，在A1事件后女性的症状表现显著高于男性（29.9 vs 15.4% PTSD诊断率），非A1事件后没有性别差异（女性28.2%，男性31.3%）。进一步的，与PTSD症状严重程度相关的焦虑和知觉到的影响在女性中高于男性。

**结论**：总的来说，女性在两类事件后的PTSD症状水平相似，男性在非A1事件后比A1事件报告更高的PTSD症状水平。这些发现说明了PTSD症状学中的性别作用和A1标准的临床有用性。

## 1. Background

Post-Traumatic Stress Disorder (PTSD) is one of only a few disorders in the DSM (American Psychiatric Association, 2013) that require an aetiological factor (a traumatic event) for its diagnosis. In the DSM-IV-TR this so-called A1 criterion involved experiencing, witnessing or being confronted with an event or events that involve actual or threatened death or serious injury, or a threat to the physical integrity of self or others (American Psychiatric Association, 2000). In the DSM-5, the A1 criterion has been narrowed to ‘exposure to actual or threatened death, serious injury or sexual violence’ (American Psychiatric Association, 2013). This means that events such as the unexpected death of a family member or a close friend due to natural causes do not meet the A1 criterion of PTSD anymore. During the last decades there has been an ongoing debate about the validity and clinical usefulness of the A1 criterion. One of the first critiques is that other (non-A1) stressful life events can also cause PTSD (Breslau & Davis, 1987). Since this influential paper, several studies have reported that stressful non-A1 events are associated with similar or even higher rates of PTSD symptoms than A1 events (e.g. Anders, Frazier, & Frankfurt, 2011; Cameron, Palm, & Follette, 2010; Gold, Marx, Soler-Baillo, & Sloan, 2005; Long et al., 2008; Mol et al., 2005; Roberts et al., 2012; Robinson & Larson, 2010), questioning the constricted definition of traumatic A1 events. In this regard, in contrast to the DSM-5, the ICD-11 will differentiate less between effects of formal DSM traumatic (A1) events and other (non-A1) stressful life events (World Health Organization), and diagnosis of PTSD will mainly be based on PTSD symptom presentation (Maercker et al., 2013; World Health Organization; Vermetten, Baker, Jetly, & McFarlane, 2016). Hence, this calls for a renewed discussion on the role of stressful life events in the development of PTSD.

Furthermore, women are approximately twice as likely to meet criteria for PTSD than men, even though women are less likely to experience an A1 event (Olff, Langeland, Draijer, & Gersons, 2007; Tolin & Foa, 2008). Men and women tend to experience different types of A1 events but, even after controlling for type of experienced A1 event, the gender differences in PTSD prevalence remain (Christiansen & Hansen, 2015; Moser, Hajcak, Simons, & Foa, 2007; Tolin & Foa, 2008). It is still unknown whether the increased vulnerability in women to develop PTSD after experiencing A1 events also extends to the experience of non-A1 events. Earlier studies that examined the association between A1 versus non-A1 events and PTSD symptom severity only investigated women (e.g. Anders et al., 2011; Cameron et al., 2010; Roberts et al., 2012) or did not investigate gender differences (e.g. Gold et al., 2005).

Little is known about the mechanisms behind gender differences in PTSD development. A possible explanation may be that women experience (A1 and non-A1) stressful events as more anxiety provoking. Anxiety sensitivity predicts PTSD symptom severity and it is suggested that this association is stronger for women (Feldner, Zvolensky, Schmidt, & Smith, 2008; Marshall, Miles, & Steward, 2010). Such peri-traumatic processes, including appraisal processes concerning the trauma, play an important role in the development of PTSD after trauma (Ozer, Best, Lipsey, & Weiss, 2003). Subjective measures of distress or impact of experienced events are often even better in predicting PTSD symptoms than objective measures of danger during events (McNally, 2003). Some studies indeed suggest that these initial responses to trauma may account for gender differences in PTSD (e.g. Irish et al., 2011), but a review by Olff et al. (2007) emphasizes that there is a serious lack of evidence on gender specific appraisal processes of trauma.

Lastly, co-morbidity between PTSD and other psychopathology is common, with the majority of PTSD patients meeting criteria for at least one other psychiatric disorder (e.g. Brady, Killeen, Brewerton, & Lucerini, 2000; Flory & Yehuda, 2015). However, to date it is unclear whether comorbid psychopathology heightens PTSD sensitivity and whether this is related to gender differences in PTSD symptoms.

The current study is the first to examine the associations between type of events and PTSD symptom severity by specifically focusing on how gender may affect the impact of those events using a large, mostly clinical sample. In 427 men and 1006 women it will be examined whether (1) non-A1 and A1 events differ regarding symptom severity and symptom domains of PTSD, (2) the link between type of event and PTSD symptoms is different for men and women, and (3) anxiety and appraisal of experienced events play a role in potential gender differences with respect to the impact of event type and PTSD symptoms.

## 2. Method

### 2.1. Study design and population

Data for the present study were drawn from the Netherlands Study of Depression and Anxiety (NESDA), an ongoing longitudinal cohort study among 2981 participants at baseline. The NESDA sample consists of individuals with a past or current depression and/or anxiety disorder, and healthy controls. General inclusion criteria were an age of 18 through 65 years during baseline assessment and being fluent in Dutch. The presence of clinically overt other psychiatric conditions that required specific other treatment (e.g. obsessive-compulsive disorder, bipolar disorder, PTSD, psychotic or severe substance use disorder) was an exclusion criterion and these disorders were not included in the NESDA study, because the primary focus of the study was on depressive and anxiety disorders (see also Spinhoven, Penninx, Van Hemert, De Rooij, & Elzinga, 2014). Since there was no active screening for PTSD, PTSD was still quite prevalent (27.8% in our sample [*n *= 398: 108 men and 290 women]; 6.7% in the total NESDA sample). The study protocol was approved centrally by the Ethical Review Board of the VU University Medical Center Amsterdam and by local review boards of each participating centre. All respondents provided written informed consent. Further details about NESDA are provided elsewhere (Penninx et al., 2008).

Four years after the baseline assessment (T4) a face-to-face assessment was conducted by trained research staff with a response rate of 80.6% (*n *= 2402), including the Life Events Checklist (LEC; see below) and a clinical interview on PTSD symptoms (PSS-I; see below). Of all participants who were interviewed with the LEC (*n *= 2402), *n *= 2165 participants indicated that they experienced an A1 or stressful non-A1 event. Of this group, *n *= 1156 participants reported an A1 event as their index event, whereas *n *= 1000 participants reported a non-A1 event.

### 2.2. Measures

#### 2.2.1. Post-traumatic stress symptoms

Administration of the PTSD Symptom Scale – Interview Version (PSS-I; Foa, Riggs, Dancu, & Rothbaum, 1993) was preceded by the Life Events Checklist (LEC; Weathers, Keane, & Davidson, 2001) in order to assess possible exposure to A1 or non-A1 events according to the DSM-IV-TR (American Psychiatric Association, 2000). The LEC describes 16 potentially traumatic A1 events and participants were asked whether they had experienced any of these events ever during their lives. Moreover, participants were asked whether they had experienced any of the following four non-A1 events (the death of someone close to you [other than sudden violent or unexpected death of someone close to you], a severe physical illness, relational problems, problems at work), and whether they had experienced any additional other impactful (A1 or non-A1) events ever in their lives. Next, participants were asked to select one of all reported (A1 and non-A1) events as their most bothersome experience (i.e. index event; ‘please select your most bothersome event from all previously mentioned events’) and when that event started and ended.

The PSS-I followed with three screening questions asking whether during the past five years (or during a shorter time period in case the event was more recent) the participant had been bothered by intrusive thoughts or images, avoidance of event related cues or heightened arousal related to the index event. When one of these three screening questions was answered positively, the full PSS-I was administered. In that case, participants were asked how often they had experienced each of the 17 criteria on the three subscales for PTSD as listed in the DSM-IV-TR (i.e. five items on re-experiencing [Cluster B], seven on avoidance/numbing [Cluster C] and five on arousal [Cluster D]) during a period of four weeks of the past five years when symptoms related to the index event were most severe.

Presence of a PTSD diagnosis was based on the DSM-IV-TR symptom criteria using the criteria of Brewin et al. (Brewin, Andrews, & Rose, 2000; Engelhard, Van Den Hout, Arntz, & McNally, 2002). A symptom was scored as present when experienced at least 2–4 times a week. This is a more conservative scoring than the scoring of Foa et al. (Foa, Cashman, Jaycox, & Perry, 1997; Foa et al., 1993) in which a symptom is scored as present if it occurred at least once a week (or less). Cronbach’s α was satisfactory-to-good: re-experiencing (0.73); avoidance/numbing (0.74); arousal (0.71); and total PSS-I scale (0.88). Sensitivity of the PSS-I has been shown to be good, namely .88 (Foa et al., 1993; Foa & Tolin, 2000).

For the current study, all events mentioned by participants in the context of the LEC (including all impactful events that were additionally mentioned) were classified as A1 or non-A1 events according to the DSM-IV-TR by two independent raters using a coding system (inter-rater reliability was high: κ = 0.86, see Appendix). The coding system consisted of the 16 A1 events of the LEC, 20 types of non-A1 events (e.g. relational problems, problems at work), and a residual ‘exclusion’ category (e.g. own psychological symptoms [e.g. burn-out, depression]), not included in the analyses. Next, participants were assigned to either the A1 or non-A1 event group according to their index event.

#### 2.2.2. Anxiety during event and perceived impact of the index event

During the PSS-I participants were also asked to indicate the degree of anxiety during the index event and the perceived impact of this event on their lives during and directly after exposure on 10-point scales ranging from ‘1’ to ‘10’ (see Spinhoven, Penninx, Krempeniou, Van Hemert, & Elzinga, 2015; Spinhoven et al., 2014).

#### 2.2.3. Psychopathology

Presence of DSM-IV-TR (American Psychiatric Association, 2000) based depressive and anxiety disorders was established using the Composite Interview Diagnostic Instrument (CIDI, version 2.1), a standardized diagnostic interview that is used worldwide for assessing psychiatric diagnoses with high inter-rater reliability, high test-retest reliability and high validity (Wittchen, 1994). We determined the five-year prevalence of depressive and anxiety disorders based on the T0, T2 and T4 assessments of the NESDA study to obtain a five-year recency diagnosis (comparable to the five-year recency PTSD diagnosis of the PSS-I): 77.9% of our sample fulfilled the criteria of an anxiety or depressive disorder during the five-year period before administration of the PSS-I (see Table 1).

**Table 1.** Demographics and PSS-I scores of all included participants (*n* = 1433) and main and interaction effects for event type and gender.

	Index event: A1 event (*n *= 573)		Index event: non-A1 event (*n *= 860)				
Variables	Men	Women	Men	Women	Main effect event	Main effect gender	Interaction event x gender
Gender distribution^a^	*n *= 162; 28.3%	*n *= 411; 71.7%	*n *= 265; 30.8%	*n *= 595; 69.2%	-	*p* < .001	n.s.
Age (in years)^b^	43.96 (13.16)	41.55 (12.29)	43.44 (12.20)	40.89 (12.58)	n.s.	*p* = .001	n.s.
Educational level (in years)	12.16 (2.89)	12.02 (3.10)	12.69 (3.38)	12.68 (3.33)	*p* = .002	n.s.	n.s.
Years since index event	11.16 (14.60)	12.18 (14.35)	9.24 (10.84)	8.70 (10.09)	*p* < .001	n.s.	n.s.
% with psychopathology^c^	71.0%	82.7%	74.0%	77.6%	n.s.	n.s.	n.s.
% meeting PTSD B, C, and D criteria^d^	15.4%	29.9%	31.3%	28.2%	*p* = .006	*p* < .001	*p* = .002
PSS-I Total score^e^	10.12 (9.82)	14.24 (11.93)	13.91 (11.39)	13.41 (10.27)	*p* = .007	*p* = .005	*p* = .005
PSS-I score: Subscale A Intrusions^e^	2.94 (3.70)	4.25 (4.12)	3.72 (3.88)	4.07 (3.73)	*p* = .03	*p* < .001	n.s.
PSS-I score: Subscale B Avoidance^e^	3.96 (4.37)	5.30 (5.21)	5.77 (5.08)	5.13 (4.60)	*p* = .001	n.s.	*p* = .006
PSS-I score: Subscale C Arousal^e^	3.22 (3.40)	4.68 (4.12)	4.36 (3.99)	4.23 (3.75)	n.s.	*p* = .005	*p* = .004
PSS-I score: Anxiety during event^e^	5.62 (3.36)	6.67 (3.19)	5.46 (3.06)	5.80 (3.03)	*p* = .006	*p* < .001	n.s.
PSS-I score: perceived impact of event^e^	8.19 (2.03)	8.83 (1.56)	8.17 (1.68)	8.45 (1.51)	*p* = .04	*p* < .001	n.s.

PSS-I = PTSD Symptom Scale – Interview version; PTSD = Post-Traumatic Stress Disorder.

^a^Gender distribution: 0 = men; 1 = women. ^b^Mean age in years during the PSS-I is reported. ^c^Psychopathology (depression and anxiety) during five years before administration of the PSS-I as measured with the CIDI is reported. ^d^PTSD B, C and D according to the Brewin & Engelhard criteria. ^e^Means and SDs of the untransformed raw PSS-I scores are reported.

## 3. Analyses

**Figure 1.**
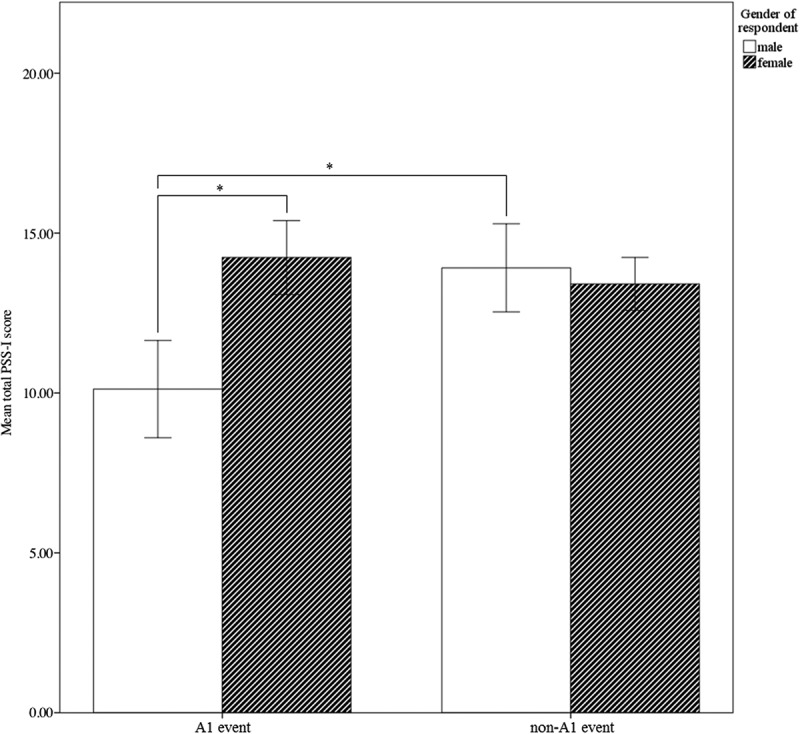
Mean total PSS-I scores for men and women per type of event. Untransformed PSS-I scores are presented.

Log PSS-I scores (PSS-I subscale and total scores) were calculated to normalize the data and were used as main outcome variables. Untransformed PSS-I scores are presented in Table 1, Figure 1 and table 2 in the Appendix.

To examine possible main effects for event type (A1 versus non-A1 events) and gender, and interaction effects between event type and gender, an ANOVA and MANOVA were conducted. Moreover, ANOVA’s were conducted to investigate the role of anxiety during and perceived impact after exposure to the index event. Statistical analyses were run using SPSS version 21 at alpha .05, with a Bonferroni correction for all analyses.

## 4. Results

### 4.1. Participants and events

Of all participants with an A1 index event, 49.6% (*n *= 573) indicated on the screening questions of the PSS-I that they were bothered by intrusive thoughts or images, avoidance of event related cues and/or heightened arousal related to the index event during the past five years (or during a shorter time period in case the event was more recent) versus 86.0% (*n *= 860) of all participants with a non-A1 index event. The complete PSS-I was administered in these cases, hence this sample was selected for the current study (*n *= 1433). See Table 1 for demographics and mean (*SD*) PSS-I scores.

The most commonly reported A1 index event for both men and women was the sudden unexpected death of someone close. A life-threatening illness or injury was the second most frequently reported A1 index event for men, whereas sexual assault was the second most commonly reported A1 event for women. Regarding non-A1 index events, both men and women reported a severe physical illness and relational problems most frequently (see Table 2 in Appendix).

### 4.2. PSS-I symptoms

The ANOVA with the PSS-I total score as dependent variable, shows a main effect for event type (*F*(1, 1429) = 7.41, *p* = .007, partial η^2^ = 0.005) and gender (*F*(1, 1429) = 7.95, *p* = .005, partial η^2^ = 0.006). Moreover, these two main effects are specified by an interaction for type of event and gender (*F*(1, 1429) = 8.02, *p* = .005, partial η^2^ = 0.006). Men and women show similar levels of PTSD symptoms after non-A1 events, whereas women show significantly higher PTSD symptoms after A1 events than men. Moreover, men show significantly higher PTSD symptoms after non-A1 events, whereas women show similar levels of PTSD symptoms after both types of events (see Figure 1).

### 4.3. PSS-I subscales

The MANOVA with the PSS-I subscale scores as dependent variables and type of event and gender as fixed factors, using Wilks’s statistic, shows similar interaction between type of event and gender with respect to avoidance (Λ = 0.99, *F*(1,1426) = 7.66, *p* = .006, partial η^2^ = 0.005) and arousal (Λ = 0.99, *F*(1,1426) = 8.18, *p* = .004, partial η^2^ = 0.006). Men whose index event was a non-A1 event report higher levels of avoidance and arousal than men whose index event was an A1 event, whereas women do not report any significant differences in avoidance or arousal after both types of events. Furthermore, participants report higher intrusion scores after experiencing non-A1 events than A1 events as index event (Λ = 0.99, *F*(1,1426) = 4.69, *p* = .03, partial η^2^ = 0.003) and women report higher intrusion scores than men (Λ = 0.99, *F*(1,1426) = 17.50, *p* < .001, partial η^2^ = 0.01). No interaction was found for intrusion scores (*p* > .05).

### 4.4. Potential confounders

We also investigated whether several possible confounders might explain the interaction effect for type of event and gender on PSS-I total scores (see Appendix for full analyses). In short, the interaction effect for type of event and gender became somewhat smaller but remained significant when we repeated our analyses leaving out all sexual assault (interaction type of event x gender: *p* = .03, partial η^2^ = 0.004). Moreover, this was also the case when adding depression/anxiety diagnoses as a predictor (main effect depression/anxiety: *p* < .001, partial η^2^ = 0.082; interaction type of event x gender: *p* = .04, partial η^2^ = 0.003), indicating that our findings cannot be explained by differences in comorbid depression and/or anxiety diagnoses. Furthermore, when we added the number of years since the event (main effect on PTSD symptoms: *p* = .76) and the number of recent negative life events in the five years preceding the administration of the PSS-I (main effect on PTSD symptoms: *p* < .001, partial η^2^ = 0.027) as covariates the interaction effect for type of event and gender remained significant (interaction type of event x gender for number of years since the event: *p* = .02, partial η^2^ = 0.004; interaction type of event x gender for number of recent negative life events: *p* = .008, partial η^2^ = 0.005). To examine whether our results are specific for events that happened a long time ago we repeated our main analysis for participants who experienced their index event in the last five years (*n *= 715). The finding that life events are at least as burdensome as A1 events holds up (no main effect for event: *p* = .11), but the finding that men report significantly more symptoms on non-A1 than A1 events is less clear for more recent events (main effect gender: *p* = .04, partial η^2^ = 0.006, but no interaction effect between type of event and gender: *p* = .50). Coding all index events according to the DSM-5 did not change our main findings either (see Appendix).

### 4.5. The role of anxiety and perceived impact

The ANOVA with gender and type of event as independent factors showed that both men and women report significantly higher levels of anxiety during exposure to A1 compared to non-A1 events (*F*(1,1428) = 7.68, *p* = .006, partial η^2^ = 0.005) and also higher levels of perceived impact after exposure to A1 compared to non-A1 events (*F*(1,1427) = 4.12, *p* = .04, partial η^2^ = 0.003; see Table 1). Overall, women report higher anxiety scores than men (*F*(1,1428) = 14.27, *p* < .001, partial η^2^ = 0.01), and also higher levels of perceived impact of the events than men (*F*(1,1427) = 22.89, *p* < .001, partial η^2^ = 0.02). There is no interaction effect between type of event and gender for the degree of anxiety (*p* = .05) nor perceived impact (*p* = .06), see Table 1.

Additionally, levels of anxiety and impact were more strongly associated with PTSD symptom severity for women (anxiety: *r* = .30, *p* < .001; impact: *r* = .31, *p* < .001) compared to men (anxiety: *r* = .19, *p* = .01; impact: *r* = .20, *p* = .01) after A1 events, but after non-A1 events associations of anxiety and impact with PTSD symptom severity were comparable for men (anxiety: *r* = .26, *p* < .001; impact: *r* = .28, *p* < .001) and women (anxiety: *r* = .32, *p* < .001; impact: *r* = .30, *p* < .001).

## 5. Discussion

### 5.1. Main findings

The DSM (American Psychiatric Association, 2013) requires the experience of a traumatic A1 event for the diagnosis of PTSD, thereby aiming to select only the most severe cases of PTSD. In contrast, in line with previous research (e.g. Anders et al., 2011; Gold et al., 2005; Mol et al., 2005) and the ICD-11 approach (World Health Organization), the current study shows in a large, mostly clinical sample that PTSD symptoms were equally or more severe in participants reporting non-A1 events than A1 events. Remarkably, 86.0% of all participants from the non-A1 event group indicated to be bothered by intrusions, avoidance of event related cues and/or heightened arousal related to the index event during the past five years versus 50% of the A1 event group. More specifically, men who experienced a non-A1 index event, such as a severe physical illness or relational problems, showed significantly higher PTSD scores than men whose index event was an A1 event, particularly in terms of avoidance and arousal symptoms. For women PTSD symptom severity was the same in both event groups. Moreover, it was striking that whereas in the A1 event group women showed significantly higher PTSD symptoms than men (29.9 versus 15.4% met PTSD B, C and D criteria), in line with previous studies (e.g. Tolin & Foa, 2008), in the non-A1 event group there were no gender differences in PTSD symptoms (women: 28.2%; men: 31.3%).

Most of the earlier studies that investigated the association between A1 versus non-A1 events and the severity of PTSD symptoms only investigated female participants or did not report on gender differences (e.g. Anders et al., 2011; Cameron et al., 2010; Roberts et al., 2012). The only study that did investigate gender differences reported that different types of traumas might be associated with differences in PTSD symptoms in women but not in men, but was limited by using a non-clinical sample and investigating a limited number of events (Lancaster, Melka, Rodriguez, & Bryant, 2014). In contrast, in the current study women did not report differences in the severity of PTSD symptoms on any of the symptom clusters per type of event, while men reported more intrusions, arousal and especially higher levels of avoidance symptom severity after non-A1 versus A1 events.

Regarding the type of reported non-A1 index events, we found that for both men and women severe physical illnesses, relational problems and the death of someone close are among the most commonly reported non-A1 index events. This is in line with previous research (e.g. Mol et al., 2005; Roberts et al., 2012). The high levels of PTSD symptoms after such events could be explained by the fact that interpersonal, relational events are particularly distressing and predictive of PTSD symptoms (Anders et al., 2011; McNally & Robinaugh, 2011), underscoring the need for a new perspective on PTSD symptoms after stressful versus traumatic life events.

We tried to examine the underlying mechanism of the gender-related differences in PTSD symptomatology. We found that comorbid anxiety and/or depression heightens PTSD sensitivity, but this was not related to gender differences in PTSD symptoms. Moreover, a higher number of recently experienced negative life events was also associated with higher levels of PTSD symptoms but this could not explain the gender differences either. Finally, we aimed to investigate whether anxiety and appraisal of non-A1 and A1 events are involved in the gender-related differences in PTSD symptomatology. Overall, participants reported significantly higher levels of anxiety and perceived impact after exposure to A1 compared to non-A1 events. Moreover, women reported higher anxiety and perceived impact of either events than men. This is in line with studies showing that women report higher levels of perceived life threat after traumatic A1 events which is predictive of posttraumatic distress (Irish et al., 2011) and might be associated with lower levels of perceived control in women compared to men after A1 events (e.g. Mak, Blewitt, & Heaven, 2004; Olff et al., 2007). Furthermore, anxiety sensitivity more strongly predicts PTSD symptom severity in women (Feldner et al., 2008). However, even though higher anxiety and perceived impact in women may partly explain the higher PTSD scores in women than in men after experiencing A1 events, this cannot explain the lack of gender differences in PTSD symptoms after non-A1 events. Moreover, this is also at odds with the finding that men experience more PTSD symptoms after non-A1 versus A1 events. Similarly, levels of anxiety and impact were more strongly associated with PTSD symptom severity for women compared to men after A1 events, but not after non-A1 events, showing differential psychological processes may underlie the development of PTSD symptoms after non-A1 versus A1 events in men and women. While the presence of comorbid depression and/or anxiety was clearly associated with higher PTSD levels, this could not explain the gender differences in PTSD symptom severity.

The use of different stress-regulating coping strategies after the experience of A1 and non-A1 events in men and women might help explain our findings. It is remarkable that men report particularly high levels of avoidance after non-A1 events compared to A1 events. Avoidance refers to cognitive, emotional, and behavioural avoidance strategies and studies show that avoidance coping is prospectively associated with PTSD symptoms (e.g. Hayes, Wilson, Gifford, Follette, & Strosahl, 1996). Given the role of gender in the socialization of emotion processing and regulation (Root & Denham, 2010), it is possible that men show more avoidance after non-A1 life events compared to A1 events because it is less socially accepted for men to be affected by events that are not officially classified as traumatic. Higher levels of peri-traumatic dissociation in men after non-A1 events might also play a role, since peri-traumatic dissociative symptoms are associated with increased PTSD risk as well (Bryant & Harvey, 2003; Fullerton et al., 2001), although we did not measure dissociation in the current study. The use of a longitudinal design is recommended for future studies to more precisely examine the potential underlying mechanisms (e.g. gender-specific coping strategies) driving the gender differences we found, while focusing on DSM-5 PTSD symptom presentation instead of the A1 criterion.

### 5.2. Strengths and limitations of our study

A main strength of the current study is the large, mostly clinical sample of 427 men and 1006 women with careful assessments of comorbid psychopathology, based on structured interviews by trained researchers. This made it possible to reliably investigate gender differences in PTSD symptom severity and to carefully investigate the role of comorbid depression and/or anxiety in the context of the gender discussion, which has not been addressed in previous studies. Moreover, given the high comorbidity between PTSD and other psychopathological conditions, specifically depression (21–94%) and other anxiety disorders (39–97%; Ginzburg, Ein-Dor, & Solomon, 2010; Perkonigg, Kessler, Storz, & Wittchen, 2000), a clinical sample as the current one is representative of the general PTSD population.

A first limitation of the current study is that participants were not explicitly asked to identify *all* experienced stressful life events so that we were not able to take into account the total number of experienced A1 and non-A1 events. Moreover, we have no specific information about the amount of time between exposure to the index event and the period of four weeks when symptoms were most severe. A next limitation is that individuals with a primary severe diagnosis of PTSD or substance use disorder (SUD) that required specific other treatment were initially omitted from the NESDA study. However, because there was no active screening for PTSD or SUDs, PTSD and SUD was still quite prevalent in our sample (PTSD: 27.8%) and in the total NESDA sample (6.7%; Boschloo et al., 2011; Manthey et al., 2012; Spinhoven et al., 2014), and therefore we expect little impact on our results. Moreover, peri-traumatic anxiety and perceived impact were measured with one-item interview questions only and future studies may profit from a more comprehensive assessment of these constructs. Furthermore, since we used a between-subject design, pre-existing differences between the A1 and non-A1 event group may have affected the outcomes as well. For instance, participants in the A1 event group had a somewhat lower educational level. However we controlled for this, and this does not seem to have affected our results. Nonetheless, there could have been other group differences we did not account for. Finally, the experience of index events and PTSD symptom severity was measured retrospectively, which may have affected the recall of events and symptoms (i.e. omission and biased retrieval) in some participants. This potential recall bias might be dependent on gender. For instance, women might report more traumatic events perpetrated by someone close, whereas men might report more events perpetrated by someone not so close (Friedrich, Talley, Panser, Fett, & Zinsmeister, 1997; Goldberg & Freyd, 2006). Again, prospective research would be important to explore this potential bias.

## 6. Conclusions

Altogether, these findings indicate that stressful life events that are not classified as traumatic, according to the DSM A1 criterion, can generate at least the same levels of PTSD symptom severity as A1 events. Several traumatic events defined as A1 events in the DSM-IV-TR (American Psychiatric Association, 2000), for example a serious illness of the self or a close friend or family member and a sudden (non-violent) unexpected death due to natural causes, were excluded in the DSM-5. As a result, some individuals who met the DSM-IV-TR symptom criteria of PTSD do not meet the DSM-5 PTSD criteria (e.g. Hoge, Riviere, Wilk, Herrell, & Weathers, 2014; Kilpatrick et al., 2013). Our study emphasizes that these stressful event types can cause similar levels, and for men even higher levels,  of symptoms and suffering in daily functioning. This questions the rationale behind these changes, and the definition of the A1 criterion in general. It is questionable whether individuals with at least as high PTSD symptom severity but no official A1 criterion should be excluded from treatment, or from reimbursement of treatment. In fact, based on the current findings and in line with the approach of the ICD-11, we recommend clinicians to pay attention to PTSD symptom profiles rather than the strict definition of the A1 criterion, to prevent highly symptomatic individuals being excluded from treatment. Furthermore, our results underscore the impact of life events in general and the adjustment problems that men and women may encounter after such life events. People report high levels of anxiety during life events and high levels of perceived impact after exposure to these life events. Moreover, a higher number of recent negative life events was also associated with higher levels of PTSD symptoms. Since negative life events are highly prevalent, studying factors associated with successful adaptation to those events could help make society more resilient and prevent stress and suffering in daily life. Frequently reported stressful life events, for example relational and work problems, seem to be on a more practical and controllable level than most A1 events such as the sudden, unexpected death of someone close. Therefore, it would be interesting to examine whether treatments for adjustment to specific types of life events, for instance focused on coaching and coping, would be more effective than exposure-based trauma treatments.

## Supplementary Material

Supplementary Material

## Appendix

### Method

#### Study design and population

A total of 673 other participants who mentioned that they experienced an A1 (*n *= 560) or non-A1 (*n *= 113) index event did not experience this event as bothersome during the last five years according to the screening questions, and therefore the other PSS-I questions were not administered. Nine participants reported several events, but did not select their index event and did not answer the screening questions. Other participants indicated that they did experience an A1 (*n *= 23) or non-A1 (*n *= 27) index event, but did not answer the screening and following PSS-I questions. Furthermore, *n *= 18 participants were excluded from further analyses because they either stated that their index event was the experience of their own psychopathology (burn-out, depression, etc.; *n *= 14) or listed an event that did not fit into the A1 or non-A1 event category (*n *= 4).

#### Measures

##### Post-traumatic stress symptoms

The list of non-A1 events of the coding system was composed based on the most frequently mentioned non-A1 events by participants to enable classification of all events into one of the three categories. Some participants (of the final participant group) mentioned more than one event as index event (*n *= 99). When an A1 event was mentioned as one of these events, they were assigned to the A1 event group. In all other cases, they were allocated to the non-A1 event group.

Correlation coefficients between PSS-I scales were as follows: re-experiencing with avoidance/numbing = 0.58; re-experiencing with arousal = 0.56; and avoidance/numbing with arousal = 0.63.

### Results

#### Potential confounders

To check whether the higher severity of PTSD symptoms for women in the A1 event group was mainly driven by higher frequency of sexual assault, we repeated our analyses leaving out all sexual assault. The interaction effect for type of event and gender remained significant (*F*(1, 1333) = 4.87, *p* = .03, partial η^2^ = 0.004). We also investigated the potential effect of five-year prevalence of psychopathology (assessed with the CIDI, see Table 1) by performing an ANOVA with the PSS-I total score as dependent variable and type of event, gender and the presence/absence of anxiety and/or depression diagnoses as fixed factors. Again, the interaction effect for type of event and gender remained significant (*F*(1, 1425) = 4.07, *p* = .04, partial η^2^ = 0.003), with psychopathology as a significant predictor (*F*(1, 1425) = 126.65, *p* < .001, partial η^2^ = 0.082). There was no three-way interaction of type of event with gender and psychopathology (*F*(1, 1425) = 0.079, *p* = .78, partial η^2^ = 0.000). These results indicate that our findings cannot be explained by differences in comorbid depression and/or anxiety diagnoses. Furthermore, non-A1 events took place more recently than the A1 events. When we added the number of years since the event as a covariate the main effect for type of event (*F*(1, 1308) = 8.49, *p* = .004, partial η^2^ = 0.006) and interaction effect for type of event and gender also remained significant (*F*(1, 1308) = 5.50, *p* = .02, partial η^2^ = 0.004). Moreover, when we added the number of negative life events in the past five years as reported on the LTE-Q (Brugha, Bebbington, Tennant, & Hurry, 1985; main effect on PTSD symptoms: *p* < .001, partial η^2^ = 0.027) as a covariate the main effect for type of event (*F*(1, 1427) = 9.27, *p* = .002, partial η^2^ = 0.006) and interaction effect for type of event and gender remained significant (*F*(1, 1427) = 6.97, *p* = .008, partial η^2^ = 0.005). There was no interaction of gender with number of recent life events (*F*(1, 1427) = 0.349, *p* = .56, partial η^2^ = 0.000). Next, to examine whether our results are specific for events that happened a long time ago, we repeated our main analysis for participants who experienced their index event in the last five years (*n* = 715; 213 men and 502 women; 279 A1 index events and 436 non-A1 index events). An ANOVA with the PSS-I total scores as dependent variable and type of event and gender as fixed factors showed a significant main effect for gender (*F*(1, 711) = 4.24, *p* = .04, partial η^2^ = 0.006; higher PSS-I scores for women), but no main effect for event (*p* = .11), nor an interaction effect between type of event and gender (*p* = .50), even though men do show higher symptoms for life events than for A1 events. The finding that life events are at least as burdensome as A1 events holds up, but the finding that men report significantly more symptoms after non-A1 than A1 events is less clear for more recent events.

In the DSM-5 the A1 event ‘sudden, unexpected death of someone close to you’ was reformulated as ‘sudden accidental death’. Additionally, the DSM-5 only qualifies sudden, catastrophic life-threatening illness or injury as an A1 event. Because the LEC was administered according to the DSM-IV-TR in the NESDA study, these details about the reported events are missing, hence we were unable to code all events according to the DSM-5. To check whether our results still hold when not including the A1 event categories from the LEC that would be modified according to the DSM-5 (‘sudden, unexpected death of someone close to you’ and ‘life-threatening illness or injury’), we repeated our analyses leaving out all participants with an index event from one of these two A1 event categories (*n = *209). An ANOVA with the PSS-I total score as dependent variable and type of event and gender as fixed factors shows that the interaction effect for type of event and gender remained significant (*F*(1, 1429) = 12.68, *p* < .001, partial η^2^ = 0.009), indicating that coding all index events according to the DSM-5 did not change our main findings.

A total of 99 individuals in the final dataset reported >1 index event. This group consisted of 24.2% men and 75.8% women, hence there are no gender differences compared to the rest of the sample (χ2 = 1.57, *p* = .21). We repeated our main analysis to check whether the results hold if these cases were omitted from the analysis. We performed an ANOVA with the PSS-I total score as dependent variable and type of event and gender as fixed factors. The main effects of gender (*p* = .007, partial η^2^ = 0.005) and type of event (*p* = .03, partial η^2^ = 0.004) as well as the interaction effect for type of event and gender remained significant (*p* = .008, partial η^2^ = 0.005).

**Table 2.** Mean total PSS-I scores of all participants for whom the PSS-I was completed.

	*n*		Mean PSS-I scores^a^	
	men	women	men	women
**A1 index events**				
Natural disaster (for example flood, hurricane, earthquake)	1	1	2.00	0.00
Fire or explosion	4	6	21.75	18.83
Transportation accident (for example car accident, train wreck, plane crash)	9	33	12.00	10.58
Serious accident at work, home or during recreational activity	8	9	10.25	10.33
Exposure to toxic substance (for example dangerous chemicals, radiation)	2	1	13.00	0.00
Physical assault (for example being attacked, hit or kicked)	14	41	6.11	18.68
Assault with a weapon (for example being shot and/or stabbed or threatened with a knife, gun, or bomb)	10	5	3.50	17.00
Sexual assault (rape, attempted rape, made to perform any type of sexual act through force or threat of harm)	8	58	17.50	21.28
Other unwanted or uncomfortable sexual experience	4	26	6.50	14.22
Combat or exposure to a war-zone (in the military or as a civilian)	2	2	3.00	12.50
Captivity (for example being kidnapped, abducted, held hostage, prisoner of war)	0	4	-	19.00
Life-threatening illness or injury	22	38	10.55	13.61
Severe human suffering	19	42	11.26	13.19
Sudden, violent death of someone close to you (for example homicide, suicide)	21	33	9.52	12.12
Sudden, unexpected death of someone close to you	38	111	10.42	11.29
Serious injury, harm or death caused by you	0	1	-	16.00
**Non-A1 index events**				
Death of someone close to you	45	127	6.04	10.09
Severe physical illness (of you or someone close to you)	67	162	13.34	11.99
Relational problems	65	129	15.38	15.95
Problems at work	51	66	20.48	16.94
Miscarriage, abortion, unfulfilled desire to have children, problems during childbirth, unwanted pregnancy	2	20	12.00	11.95
Death of someone not close to you (for example client, student)	1	1	15.00	2.00
Family problems: decreased contact	2	8	11.50	7.13
Family problems: psychological problems	6	13	14.50	13.62
Family problems: rest	13	29	11.77	14.90
Family problems: divorce of parents	4	9	7.25	9.33
Non-family problems: decreased contact	0	1	-	9.00
Non-family problems: psychological problems	1	3	0.00	17.33
Non-family problems: rest	1	4	7.00	19.00
Financial problems	0	3	-	10.33
Burglary, housebreaking	1	2	32.00	22.50
Moving	0	2	-	25.00
Bullying and stalking	3	10	24.33	17.60
Being threatened or threatening of someone close to you	1	1	20.00	22.00
Emotional neglect	0	1	-	26.00
Psychological and emotional abuse	2	4	7.00	25.50
**Rest**				
Psychological symptoms of the participant (for example burn-out, depression)	10	4	19.53	16.75

PSS-I: PTSD Symptom Scale – Interview version.

^a^Means of the original PSS-I scores are reported.
