# Supplementary material for: A new perspective on PTSD symptoms after traumatic vs stressful life events and the role of gender
Source: Eur J Psychotraumatol. 2017 Nov 13;8(1):1380470. doi: 10.1080/20008198.2017.1380470 (PMC5800737; doi:10.1080/20008198.2017.1380470)
Supplement: Supplementary Material [file ZEPT_A_1380470_SM5647.docx]

**Appendix**

**Method**

***Study design and population***

673 other participants who mentioned that they experienced an A1 (*n*=560) or non-A1 (*n*=113) index event did not experience this event as bothersome during the last 5 years according to the screening questions, and therefore the other PSS-I questions were not administered. 9 participants reported several events, but did not select their index event and did not answer the screening questions. Other participants indicated that they did experience an A1 (*n*=23) or non-A1 (*n*=27) index event, but did not answer the screening and following PSS-I questions. Furthermore, *n*=18 participants were excluded from further analyses because they either stated that their index event was the experience of their own psychopathology (burn-out, depression etc.; *n*=14) or listed some other event that did not fit into the A1 or non-A1 event category (*n*=4).

***Measures***

*Post-traumatic stress symptoms*

The list of non-A1 events of the coding system was composed based on the most frequently mentioned non-A1 events by participants to enable classification of all events into one of the three categories. Some participants (of the final participant group) mentioned more than one event as index event (*n*=99). When an A1 event was mentioned as one of these events they were assigned to the A1 event group. In all other cases they were allocated to the non-A1 event group.
 Correlation coefficients between PSS-I scales were as follows: re-experiencing with avoidance/numbing = 0.58; re-experiencing with arousal = 0.56; and avoidance/numbing with arousal = 0.63.

**Results**

***Potential confounders***

To check whether the higher severity of PTSD symptoms for women in the A1 event group was mainly driven by higher frequency of sexual assault, we repeated our analyses leaving out all sexual assault. The interaction effect for type of event and gender remained significant (F(1, 1333) = 4.87, p = 0.03, partial η² = 0.004). We also investigated the potential effect of 5-year prevalence of psychopathology (assessed with the CIDI, see Table 1) by performing an ANOVA with the PSS-I total score as dependent variable and type of event, gender and the presence/absence of anxiety and/or depression diagnoses as fixed factors. Again, the interaction effect for type of event and gender remained significant (F(1, 1425) = 4.07, p = 0.04, partial η²= 0.003), with psychopathology as a significant predictor (F(1, 1425) = 126.65, p < 0.001, partial η²= 0.082). There was no three-way interaction of type of event with gender and psychopathology (F(1, 1425) = 0.079, p = 0.78, partial η² = 0.000). These results indicate that our findings cannot be explained by differences in comorbid depression and/or anxiety diagnoses. Furthermore, non-A1 events took place more recently than the A1 events. When we added the number of years since the event as a covariate the main effect for type of event (F(1, 1308) = 8.49, p = 0.004, partial η² = 0.006) and interaction effect for type of event and gender also remained significant (F(1, 1308) = 5.50, p = 0.02, partial η² = 0.004). Moreover, when we added the number of negative life events in the past 5 years as reported on the LTE-Q (Brugha, Bebbington, Tennant, & Hurry, 1985; main effect on PTSD symptoms: p < 0.001, partial η² = 0.027) as a covariate the main effect for type of event (F(1, 1427) = 9.27, p = 0.002, partial η²= 0.006) and interaction effect for type of event and gender remained significant (F(1, 1427) = 6.97, p = 0.008, partial η²= 0.005). There was no interaction of gender with number of recent life events (F(1, 1427) = 0.349, p = 0.56, partial η² = 0.000). Next, to examine whether our results are specific for events that happened a long time ago, we repeated our main analysis for participants who experienced their index event in the last five years (n=715; 213 men and 502 women; 279 A1 index events and 436 non-A1 index events). An ANOVA with the PSS-I total scores as dependent variable and type of event and gender as fixed factors showed a significant main effect for gender (F(1, 711) = 4.24, p = 0.04, partial η² = 0.006; higher PSS-I scores for women), but no main effect for event (p = 0.11), nor an interaction effect between type of event and gender (p = 0.50), even though men do show higher symptoms for life events than for A1 events. So the finding that life events are at least as burdensome as A1 events holds up, but the finding that men report significantly more symptoms after non-A1 than A1 events is less clear for more recent events.
 In the DSM-5 the A1 event ‘*sudden, unexpected death of someone close to you*’ was reformulated as ‘*sudden accidental death*’. Additionally, the DSM-5 only qualifies sudden, catastrophic life-threatening illness or injury as an A1 event. Because the LEC was administered according to the DSM-IV-TR in the NESDA study, these details about the reported events are missing, hence we were unable to code all events according to the DSM-5. To check whether our results still hold when not including the A1 event categories from the LEC that would be modified according to the DSM-5 (‘*sudden, unexpected death of someone close to you*’ and ‘*life-threatening illness or injury*’), we repeated our analyses leaving out all participants with an index event from one of these two A1 event categories (*n=*209). An ANOVA with the PSS-I total score as dependent variable and type of event and gender as fixed factors shows that the interaction effect for type of event and gender remained significant (F(1, 1429) = 12.68, p < 0.001, partial η² = 0.009), indicating that coding all index events according to the DSM-5 did not change our main findings.
 99 individuals in the final dataset reported more than 1 index event. This group consisted of 24.2% men and 75.8% women, hence there are no gender differences compared to the rest of the sample (χ2 = 1.57, p = 0.21). We repeated our main analysis to check whether the results hold if these cases were omitted from the analysis. We performed an ANOVA with the PSS-I total score as dependent variable and type of event and gender as fixed factors. The main effects of gender (p = 0.007, partial η² = 0.005) and type of event (p = 0.03, partial η² = 0.004) as well as the interaction effect for type of event and gender remained significant (p = 0.008, partial η² = 0.005).

Table 2

*Mean total PSS-I scores of all participants for whom the PSS-I was completed*

|  | N | | Mean PSS-I scores^a^ | | |
| --- | --- | --- | --- | --- | --- |
|  | men | women | men | women | |
| **A1 index events** | | | | | |
| Natural disaster (for example flood, hurricane, earthquake) | 1 | 1 | 2.00 | 0.00 | |
| Fire or explosion | 4 | 6 | 21.75 | 18.83 | |
| Transportation accident (for example car accident, train wreck, plane crash) | 9 | 33 | 12.00 | 10.58 | |
| Serious accident at work, home or during recreational activity | 8 | 9 | 10.25 | 10.33 | |
| Exposure to toxic substance (for example dangerous chemicals, radiation) | 2 | 1 | 13.00 | 0.00 | |
| Physical assault (for example being attacked, hit or kicked) | 14 | 41 | 6.11 | 18.68 | |
| Assault with a weapon (for example being shot and/or stabbed or threatened with a knife, gun, or bomb) | 10 | 5 | 3.50 | 17.00 | |
| Sexual assault (rape, attempted rape, made to perform any type of sexual act through force or threat of harm) | 8 | 58 | 17.50 | 21.28 | |
| Other unwanted or uncomfortable sexual experience | 4 | 26 | 6.50 | 14.22 | |
| Combat or exposure to a war-zone (in the military or as a civilian) | 2 | 2 | 3.00 | 12.50 | |
| Captivity (for example being kidnapped, abducted, held hostage, prisoner of war) | 0 | 4 | - | 19.00 | |
| Life-threatening illness or injury | 22 | 38 | 10.55 | 13.61 | |
| Severe human suffering | 19 | 42 | 11.26 | 13.19 | |
| Sudden, violent death of someone close to you (for example homicide, suicide) | 21 | 33 | 9.52 | 12.12 | |
| Sudden, unexpected death of someone close to you | 38 | 111 | 10.42 | 11.29 | |
| Serious injury, harm or death caused by you | 0 | 1 | - | 16.00 | |
| **Non-A1 index events** | | | | | |
| Death of someone close to you | 45 | 127 | 6.04 | 10.09 |  |
| Severe physical illness (of you or someone close to you) | 67 | 162 | 13.34 | 11.99 |  |
| Relational problems | 65 | 129 | 15.38 | 15.95 |  |
| Problems at work | 51 | 66 | 20.48 | 16.94 |  |
| Miscarriage, abortion, unfulfilled desire to have children, problems during childbirth, unwanted pregnancy | 2 | 20 | 12.00 | 11.95 |  |
| Death of someone not close to you (for example client, student) | 1 | 1 | 15.00 | 2.00 |  |
| Family problems: decreased contact | 2 | 8 | 11.50 | 7.13 |  |
| Family problems: psychological problems | 6 | 13 | 14.50 | 13.62 |  |
| Family problems: rest | 13 | 29 | 11.77 | 14.90 |  |
| Family problems: divorce of parents | 4 | 9 | 7.25 | 9.33 |  |
| Non-family problems: decreased contact | 0 | 1 | - | 9.00 |  |
| Non-family problems: psychological problems | 1 | 3 | 0.00 | 17.33 |  |
| Non-family problems: rest | 1 | 4 | 7.00 | 19.00 |  |
| Financial problems | 0 | 3 | - | 10.33 |  |
| Burglary, housebreaking | 1 | 2 | 32.00 | 22.50 |  |
| Moving | 0 | 2 | - | 25.00 |  |
| Bullying and stalking | 3 | 10 | 24.33 | 17.60 |  |
| Being threatened or threatening of someone close to you | 1 | 1 | 20.00 | 22.00 |  |
| Emotional neglect | 0 | 1 | - | 26.00 |  |
| Psychological and emotional abuse | 2 | 4 | 7.00 | 25.50 |  |
| **Rest** | | | | |  |
| Psychological symptoms of the participant (for example burn-out, depression) | 10 | 4 | 19.53 | 16.75 |  |

PSS-I: PTSD Symptom Scale – Interview version
a. Means of the original PSS-I scores are reported
